# Perforating foreign body causing incomplete facial paralysis^☆^

**DOI:** 10.1016/j.tcr.2020.100370

**Published:** 2020-11-06

**Authors:** Till Berk, Hans-Christoph Pape, Gerrolt Nico Jukema

**Affiliations:** Division of Traumatology, University Hospital Zurich, University of Zurich, Zurich, Switzerland

**Keywords:** Facial muscle weakness, Facial nerve trauma, Facial paralysis, Foreign body, Perforating injuries, Traumatic facial paralysis

## Abstract

Facial nerve trauma is a common cause of facial paralysis; both blunt and penetrating forces may compromise the facial nerve. A comprehensive primary and secondary survey is essential for diagnosis and treatment of the injury. Here we report on a young patient who sustained a quad bike accident, leading to an perforating injuries of the face from a bough, causing facial paralysis.

## Introduction

Traumatic facial paralysis is most often associated with perforating injuries to the face and fractures of the temporal bone; it is the second most common cause of a facial paralysis. Depending on its location, the injury may result in serious consequences such as ipsilateral facial muscle weakness, hyperacusis, lacrimal/salivary gland dysfunction or dysgeusia and can have a strong emotional impact on patients [1]. The timing of treatment in traumatic facial paralysis can have a significant impact on functional outcome [2,3]. Therefore, it is critical for otolaryngologists and facial plastic surgeons to have a thorough understanding of the principles of facial nerve trauma detection and management [1,4,5]. There are many possible treatment options, but initially it is vital to deliberate carefully at what time any surgical or conservative treatment should be performed [1,[6], [7], [8], [9]].

Here we report on a patient who sustained an unusual, belatedly recognized traumatic facial paralysis. To our knowledge, no similar case has yet been published.

## Case report

**Fig. 1:**
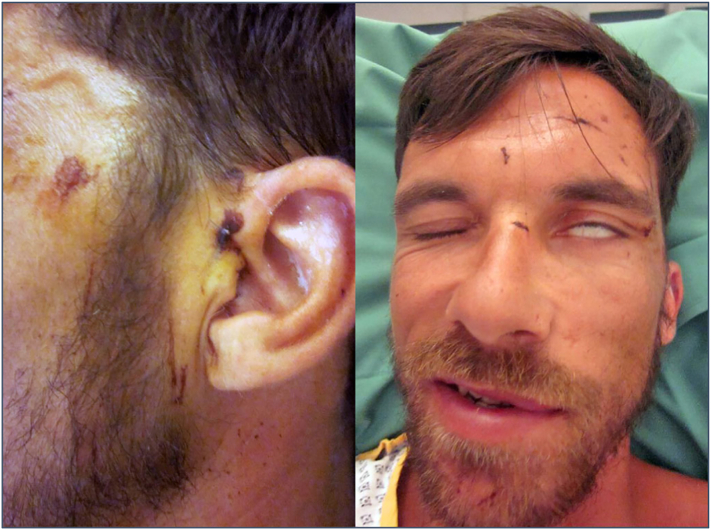
At primary survey, the patient presented with incomplete, peripheral, left sided facial paralysis (right) Four days prior to admission, the minor wound adjacent to the left tragus had been closed prematurely (left).

A 34-year-old man was transferred to our institution four days after a quad bike accident in rural Greece. Among other minor injuries, the patient had suffered a small wound posterior to the tragus of his left ear (Fig. 1). Primary closure of the wound was performed at a local infirmary; no radiological investigations had been performed at the time. Ninety-six hours later the patient presented at our hospital with obvious signs of left sided peripheral facial paralysis (Fig. 1). The aforementioned wound was slightly painful on palpation but appeared inconspicuous otherwise. When questioned about the circumstances of the accident, the patient explained that one wheel of the quad bike had become detached at full speed, catapulting him into low growing brushwood. According to the patient, he had noted (but quickly dismissed) a drooping of his left corner of the mouth shortly after the accident; the patient stated to be free of other symptoms and to be otherwise healthy. (For a details systematic facial palsy assessment, see Table 1 [10]) Upon admission at our hospital, a CT scan of the head was performed to rule out intracranial hemorrhage and fractures of the skull. These images raised suspicion of a foreign body deep to the left auricular concha (Fig. 3). No other pathologies were found; in particular, there was no indication of a fracture of the temporal bone. The further non-surgical management included concussion monitoring and wound care. An MRI scan was subsequently ordered to assess the integrity of the left facial nerve and its relation to the aforementioned foreign body. The MRI scan finally confirmed the presence of a cylindrical foreign body with surrounding edema in the immediate vicinity of the temporal styloid process and the course of the left facial nerve (Fig. 3).

**Table 1:** Systematic facial palsy assessment.

Are the ears clear on otoscopy?	• Assess the tympanic membrane and ear canal; acute or chronic otitis media (±cholesteatoma) and malignant otitis externa can all cause lower motor neurone facial palsy and will require further urgent assessment in secondary care (ear, nose, throat)
Is there ipsilateral hearing loss?	• Perform Weber’s and Rinne’s tuning fork tests; although Ramsay Hunt syndrome will be associated with sensorineural hearing loss (SNHL), it is important to exclude a cerebellopontine angle lesion (normally presents with gradual onset unilateral SNHL); acute otitis media or cholesteatoma may be associated with conductive hearing loss, whereas patients with Bell’s palsy normally have abnormal sensitivity to loud sounds
Is there a rash?	• Small vesicular eruptions that affect the tympanic membrane, ear canal, external pinna, or oral cavity may indicate Ramsay Hunt syndrome; Lyme disease is restricted to heavily forested regions and presents with a characteristic erythematous “bullseye” lesion on the limbs or trunk (70% of cases), arthralgia, and facial swelling; it is caused by a bite from a tick carrying Borrelia burgdorferi (US) or B afzalii/garinii (Europe), and an antibody test may help confirm the diagnosis
Are there any bruises or scars in the head and neck region?	• The palsy may be secondary to a skull base fracture (common associated signs include periorbital or mastoid bruising, blood in the ear canal, and haemotympanum) or recent mastoid, parotid, or submandibular gland surgery
Is the corneal reflex intact on both sides	• In addition to facial muscle weakness, a cerebellopontine angle lesion (such as an acoustic neuroma) may cause a reduced or absent corneal reflex on the affected side ± aural fullness (owing to trigeminal nerve deficit)
Is the mastoid region tender or swollen?	• A tender swelling of the mastoid with associated middle ear inflammation or pinna lateralisation (or both) may suggest acute mastoiditis
Is the parotid gland enlarged?	• Palpation of a parotid lump may suggest cancer (particularly if associated with a history of regional skin cancer, delayed onset facial palsy, or pain); if cancer is suspected, thoroughly examine the rest of the head and neck and refer urgently (for example, through the “two week wait suspected head and neck cancer pathway” in the UK)

Adapted from Masterson, Liam, et al. *Assessment and management of facial nerve palsy*. bmj, 2015, 351. Jg., S. h3725.

**Fig. 2:**
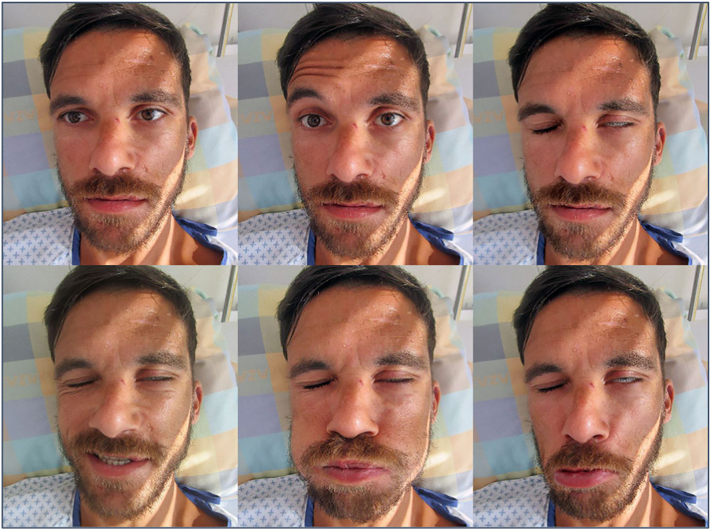
Preoperative clinical testing. The patient demonstrated incomplete, peripheral, left sided facial paralysis.

**Fig. 3:**
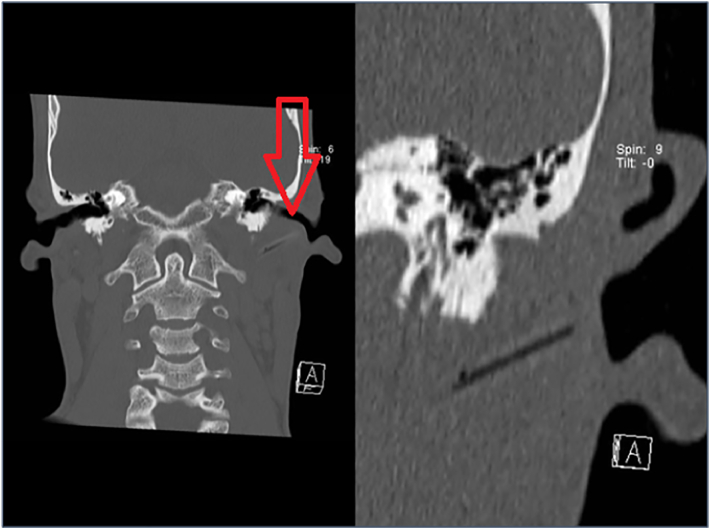
Representative coronary slices of the initial CT scan. The arrow indicates the cylindrical foreign body close to the temporal styloid process. An additional MRI was performed to identify the exact location, composition and size of the foreign body. Intraoperative Findings. After surgical exploration, a piece of wood measuring 30 × 3 mm was readily extracted.

Over the period of four days, some recovery of facial nerve function was noted clinically; however, facial expression remained strikingly asymmetrical. Regular moisturization of the left eye was necessary due to persistent lagophthalmos (Fig. 2). There was no evidence of facial nerve discontinuity. Written informed consent was obtained for exploration of the auricular lesion and foreign body removal. Superficial wound dissection revealed a piece of wood measuring 30 × 3 mm; it was readily extracted using a pair of forceps (Fig. 3). A second CT scan of the head was performed thereafter to verify complete foreign body removal. Facial nerve function presently improved and the patient was discharged home two days postoperatively. When the patient was seen again six weeks later, he was back at work and free of complaints with full recovery of facial nerve function.

## Discussion

In our case, the patient presented with incomplete, peripheral, left sided facial paralysis. Four days prior to admission, a minor wound adjacent to the left tragus had been closed prematurely, overlooking a foreign body seated deep to the point of entry and compressing the facial nerve after its emergence from the stylomastoid foramen. Upon presentation at our institution, the auricular wound was unobtrusive and the presence of foreign body was not immediately suspected. Disregarding radiographic results, one might be tempted to proceed in an observant manner considering the incompleteness and early partial recovery of facial paralysis in this patient. In the presence of a foreign body, such an approach would naturally entail the risk of infection, granuloma formation and persistent facial nerve dysfunction.

Facial nerve trauma is a relatively common cause of facial paralysis. The facial nerve may be compromised by blunt or penetrating forces anywhere along its winding course from the central nuclei to the most peripheral parts of its intratemporal and extratemporal branches [1,11,12]. Aside from iatrogenic injuries following salivary gland and otologic surgery, traumatic facial paralysis is most frequently related to fractures of the petrous bone, which may lead to disruption of the facial nerve within the Fallopian canal; by contrast, the presence of facial wounds may hint at an injury to extratemporal branches [1,5,13,14]. Due to the multitude of functional components comprised by the facial nerve, there is a wide range of symptoms following its impairment; facial muscle weakness may be the most evident feature in clinical practice, yet it is equally important to assess auditory, gustatory and lacrimal function in order to gauge possible damage to the nerve along (or proximal to) its intertemporal passage [1,13,15]. While radiological investigations such as high-resolution computed tomography are usually necessary to accurately determine the etiology of traumatic facial paralysis and to rule out concomitant intracranial injuries, clinical history taking and examination are paramount to developing a treatment plan. This is because the management of traumatic facial paralysis ultimately depends on both the onset and the evolution of symptoms [1,8,16,17]. While delayed or incomplete loss function may hint at compression or traction injury to a nerve that is anatomically intact, immediate and complete dysfunction suggests loss of neural continuity. Whereas the former symptomatology may be addressed conservatively due to exceedingly high rates of spontaneous recovery [1,18], the latter presentation may necessitate surgical exploration of the facial nerve. Depending on the findings, operative treatment may consist of nerve decompression, end-to-end neurorrhaphy or interposition nerve grafting. When in doubt, periodic electrophysiologic testing may be warranted to determine the degree of axonal degeneration and to estimate the probability of recovery [1,19]. To the best of our knowledge, a similar case to ours could be found mainly in children. In particular a foreign body resulting in chronic otomastoiditis and facial palsy in a 5-year-old girl [20].

## Conclusion

In view of the case at hand, which to our knowledge is the first of its kind to be published, we advise that a high degree of clinical suspicion be upheld in patients with incomplete traumatic facial paralysis; routine CT imaging is suggested to identify reversible causes of traumatic facial paralysis such as the presence of a foreign body even if the history is not forthcoming.

## Consent

Informed consent was obtained from the patient for publication of this case report and accompanying images.

## Declaration of competing interest

The authors report no conflict of interest concerning the materials or methods referred in this case report or the findings specified in this article. The authors state that this work has not been previously published in whole or in part or submitted elsewhere for review.
